# Supplementary material for: Solution-Processed SnO2 Quantum Dots for the Electron Transport Layer of Flexible and Printed Perovskite Solar Cells
Source: Nanomaterials (Basel). 2022 Jul 29;12(15):2615. doi: 10.3390/nano12152615 (PMC9370154; doi:10.3390/nano12152615)
Supplement: Supplementary file 1 [file nanomaterials-12-02615-s001.zip › nanomaterials-1788920-supplementary.pdf]

Supporting Information

Solution-Processed SnO_2 Quantum Dots for the Electron Transport Layer of Flexible and Printed Perovskite Solar Cells

Muhammad Salman Kiani [1], Zhandos T. Sadirkhanov [1], Alibek G. Kakimov [1], Hryhorii P. Parkhomenko [1], Annie Ng [2], and Askhat N. Jumabekov [1,*]

[1] Department of Physics, Nazarbayev University, Nur-Sultan 010000, Kazakhstan; muhammad.kiani@nu.edu.kz (M.S.K.); zhandos.sadirkhanov@nu.edu.kz (Z.T.S.); alibek.kakimov@gmail.com (A.G.K.); hryhorii.parkhomenko@nu.edu.kz (H.P.P.)

[2] Department of Electrical and Computer Engineering, Nazarbayev University, Nur-Sultan 010000, Kazakhstan; annie.ng@nu.edu.kz

* Correspondence: askhat.jumabekov@nu.edu.kz

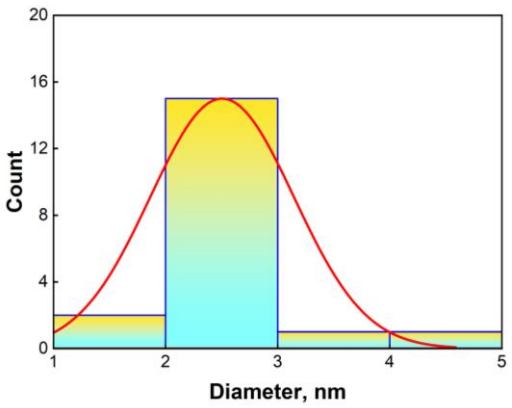

Figure S1. Particle size distribution.

Crystallite size calculation:

The Scherrer's equation [1,2]:

$$D = \frac{n\lambda}{\beta cos\theta} \quad \text{(S1)}$$

where D is crystallite size, n is the Scherrer's constant (mostly taken as equal to 0.9), λ is the wavelength of incident beam (1.54 Å), β is the full width at half-maximum (FWHM) of the peak, and θ is the Bragg angle.

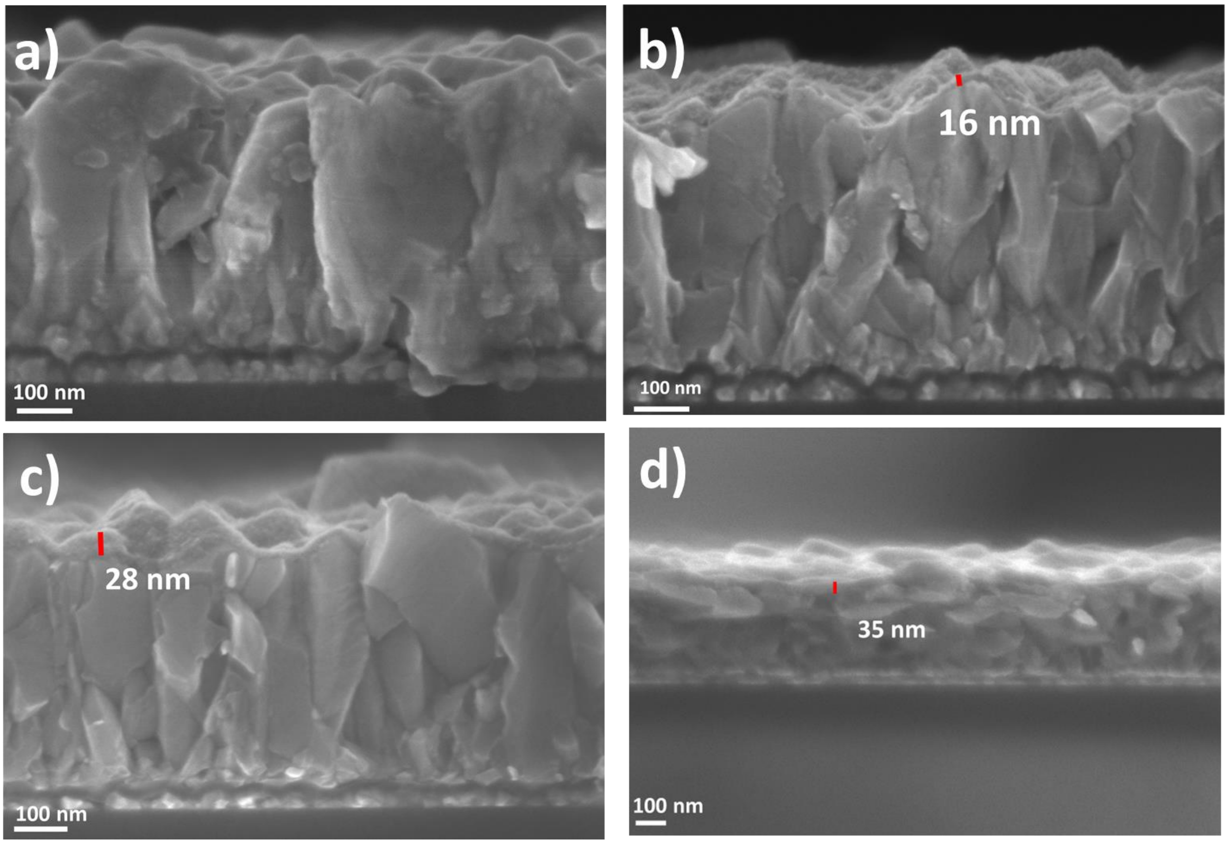

Figure S2. SEM cross-section images of ETLs on glass/FTO made with (**a**) 1 wt%, (**b**) 1.5 wt%, (**c**) 2.5 wt% and, (**d**) 3 wt% of SnO_2 QD inks.

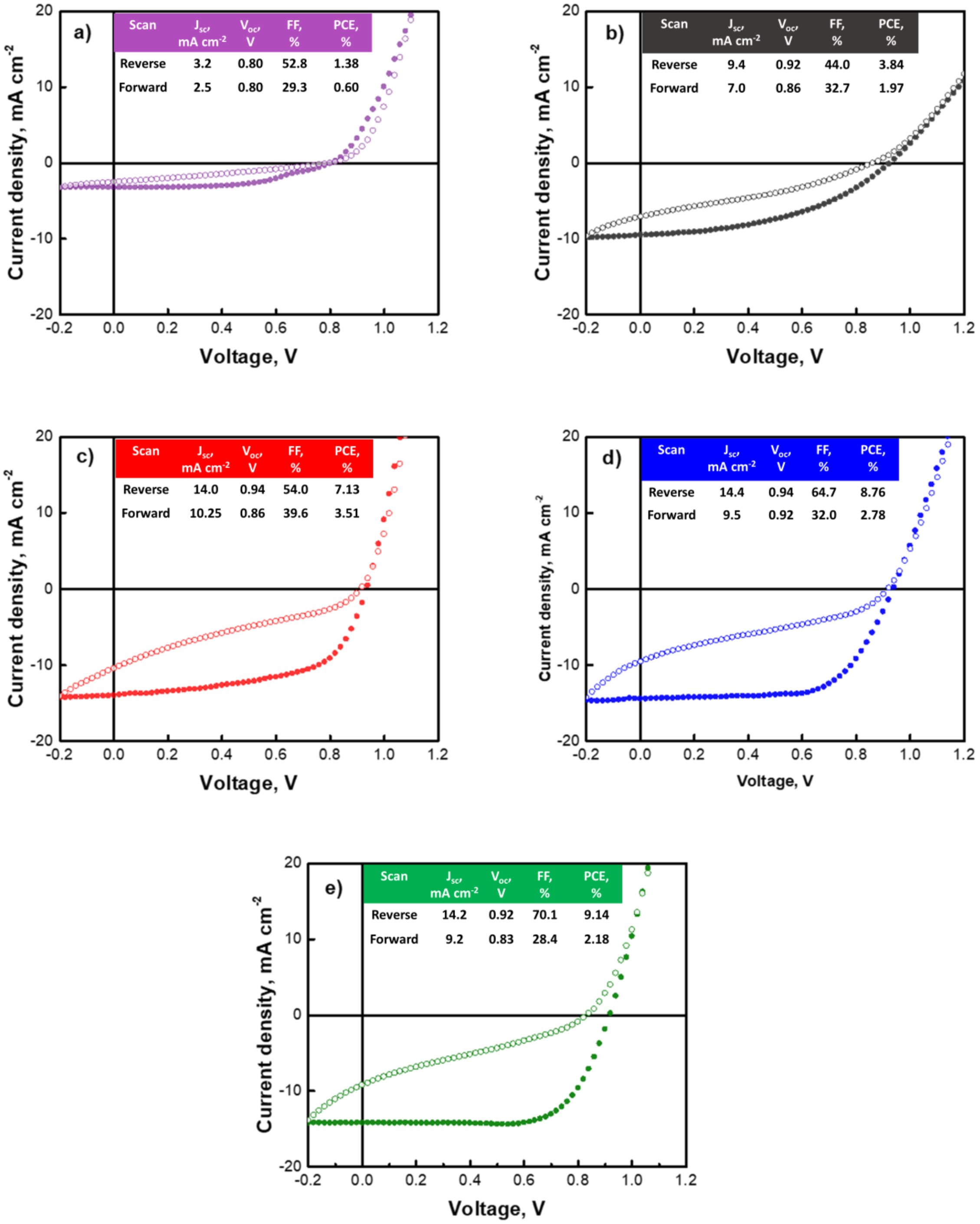

Figure S3. *J-V* curves of champion devices with (**a**) E0, (**b**) E1, (**c**) E2, (**d**) E4, and (**e**) E5 ETLs.

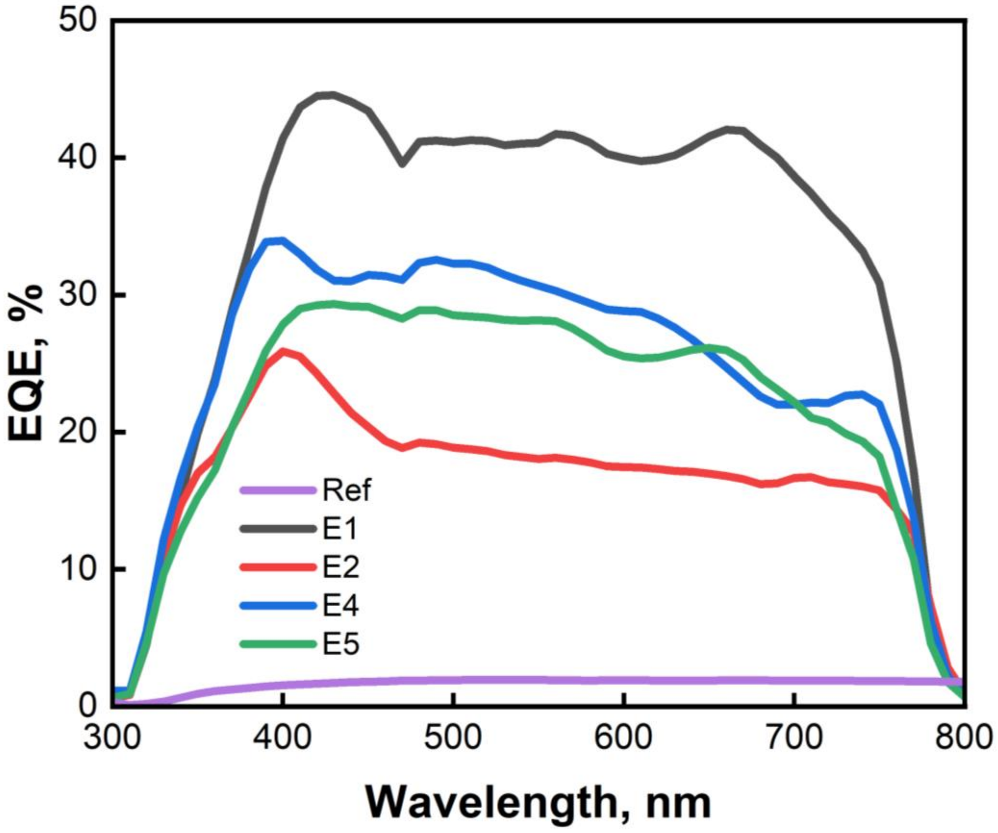

Figure S4. EQE spectra of champion devices with different ETLs (E0, E1, E2, E4, and E5).

References

1. Patterson, A.L. The Scherrer Formula for X-Ray Particle Size Determination. *Phys. Rev.* **1939**, *56*, 978–982, doi:10.1103/PhysRev.56.978.
2. Bokuniaeva, A.O.; Vorokh, A.S. Estimation of Particle Size Using the Debye Equation and the Scherrer Formula for Polyphasic TiO_2 Powder. *Journal of Physics* **2019**, 7.
